# Primary hepatic melanoma: A case report of computed tomography and magnetic resonance imaging findings

**DOI:** 10.1097/MD.0000000000016165

**Published:** 2019-06-21

**Authors:** Weiqun Ao, Jian Wang, Guoqun Mao, Guangzhao Yang, Xiaoyu Han, Yuzhu Jia, Yougen Cheng

**Affiliations:** a Department of Radiology; b Department of Pathology, Tongde Hospital of Zhejiang Province, Hangzhou, Zhejiang, China.

**Keywords:** hepatic tumors, magnetic resonance imaging, melanoma, tomography, x-ray compute

## Abstract

**Rationale:**

Malignant melanoma predominantly develops in middle-aged and older adults, most commonly occurring on the skin and rarely on internal organs. Malignant melanoma originating in the liver is extremely rare. Imaging findings of primary hepatic melanoma (PHM) are scarce in relevant literature.

**Patient concerns:**

The patient was a 69-year-old woman from Zhejiang, China, who was admitted to the hospital because of upper abdominal pain that persisted for >10 days.

**Diagnoses:**

Computed tomography (CT) findings indicated the presence of a circular low-density shadow of approximately 7.5 × 8.0 cm in the hepatic hilar region. Magnetic resonance imaging (MRI) indicated a heterogeneous solid cystic mass in the hepatic hilar region. The mass exhibited heterogeneous low-signal intensity on a T1-weighted image (T1WI) and slightly higher signal intensity on a T2-weighted image (T2WI). The tumor appeared as multiple irregular strips with high-signal intensity on T1WI and low-signal intensity on T2WI. The diffusion-weighted image revealed increased signal intensity. The tumor continued to be enhanced after enhancement. Clinical data suggested that the tumor was a malignant liver tumor.

**Interventions:**

The patient underwent a CT guide puncture hepatic biopsy. The tumor was located in the hepatic hilar region adjacent to the large blood vessels and invaded the portal vein. Because a resection was highly risky, conservative treatment was conducted.

**Outcomes:**

Postoperative pathology and clinical examination confirmed that the tumor was malignant PHM. The patient has been followed up for 6 months. The patient underwent CT reexamination 2 months after conservative treatment, the results of which revealed that the tumor progressed. Multiple lesions were identified; moreover, the tumor size had increased and the tumor had invaded the portal vein and intrahepatic bile duct. The patient was reexamined by CT in another hospital 6 months after conservative treatment. The results revealed peritoneal, omental metastases and multi bone metastases.

**Lessons:**

To our best knowledge, this is the first reported case of a PHM with complete imaging data, including preoperative CT and MRI examinations and a follow-up CT examination. From compiling the CT and MRI findings of this patient and those of relevant studies, this study can serve as a reference for the preoperative diagnosis and differential diagnosis of PHM.

## 1 Introduction

Malignant melanoma refers to a malignant tumor that has developed from melanoma cells. Studies in pathology and molecular diagnostics have revealed that malignant melanoma is a relatively rare malignant tumor with highly aggressive biological behavior. Moreover, a malignant melanoma is not a single entity; rather, it is a group of different neoplasms with variable etiopathogenesis, biological behavior, and prognosis.^[1]^ In recent decades, the incidence of melanoma in men and women has increased drastically in almost every country.^[2,3]^ Melanoma primarily develops at the junction of the epidermis and dermis, or areas rich in melanocytes, such as the skin, mucous membranes, the ciliary body of the eyeballs, the iris, choroids, and meninges.^[4]^ The risk of recurrence and metastasis of malignant melanoma is high, and the prognosis is usually poor. Metastatic spread^[5–7]^ of abdominal melanoma to the liver is common, and primary melanoma is extremely rare.^[8]^ Primary hepatic melanoma (PHM) cannot be easily identified through clinical observation and imaging technology because of its unspecific features; thus, PHM may be diagnosed as other types of hepatic malignant tumors. This study provides a report of a patient with PHM that was confirmed through clinical observation and pathological diagnosis. Patient computed tomography (CT) and magnetic resonance imaging (MRI) characteristics were analyzed, and relevant studies were reviewed to improve the understanding of PHM and provide an example that may serve as a reference for clinical treatment.

## 2 Case presentation

The patient was a 69-year-old woman who was admitted to the hospital because of upper abdominal pain that persisted for >10 days. The pain was characterized as primary distension pain without obvious cause. The patient received a B ultrasound examination at a local hospital, and the results revealed that the patient had multiple space-occupying lesions on the liver. She was treated in the Division of Gastroenterology at the author's affiliated hospital. A physical examination revealed no apparent jaundice; superficial lymph nodes, such as those on the neck, supraclavicular lymph nodes, and axillary lymph nodes were not swollen. Breath sounds were normal, and no coarse rhonchi and moist rales were noted. The patient's heart rate was 83 bpm, and heart sounds were normal. The abdomen was slightly bulging; the spleen was not palpable below the costal margin. Tenderness was reported in the right epigastrium, bowel sounds were 4 times per minute, and no shifting dullness sound or lower-limb edema were noted. A laboratory examination indicated that tumor indices CA199, CEA, AFP, and CA125 were in the normal range. A routine blood count yielded the following results: leukocyte count = 5.2 × 10^9^ cells/L, neutrophils = 69.5%, erythrocyte count = 3.37 × 10^12^ cells/L, hemoglobin = 97 g/L, total protein = 68.4 g/L, albumin = 36.5 g/L, alkaline phosphatase = 139 U/L, gamma-glutamyl transferase = 49 U/L. Leukocyte, neutrophil, and high-sensitivity C-reactive protein levels were all within the normal range. The patient had no history of hepatitis B.

The patient underwent CT and MRI examinations during hospitalization. CT was performed using a 64-MDCT scanner (Definition AS, Siemens, Germany). The parameters for both plain and enhanced CT examination were: tube voltage, 120∼130 kV; tube current 210 mAs used as the quality reference for an online dose modulation system (CareDose 4D, Siemens) for the definition scanners; slice thickness 1.5 mm. MRI was performed using a 3.0 Tesla (T) Siemens scanner (Siemens Magnetom Verio; Siemens Medical Systems, Erlangen, Germany) with a phased-array body coil. MRI sequences included T1-weighted, axial and coronal T2-weighted image; diffusion-weighted image (DWI) findings were obtained using the following parameters: echo planar imaging technique b values: 0, 400, 1000 s/mm^2^. Dynamic MR images were acquired through the fat-suppressed 3-dimensional gradient-echo sequence.

**Figure 1:**
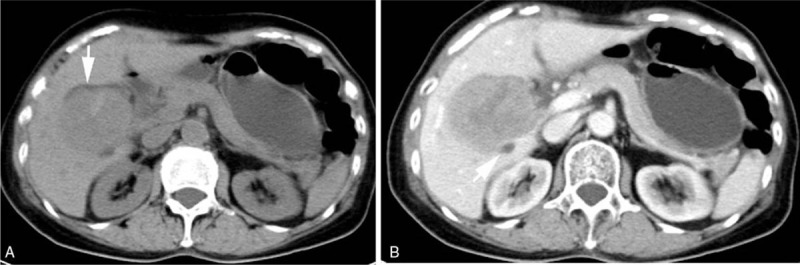
Computed tomography imaging revealed a circular low-density shadow of approximately 7.5 × 8.0 cm in the hepatic hilar region. The interior of the tumor was uneven, and the strip demonstrated high density. (A) The tumor was unevenly enhanced during scanning of the portal venous phase, and the internal density was uneven. (B) The mass compressed the intrahepatic bile duct and caused dilatation (arrow).

**Figure 2:**
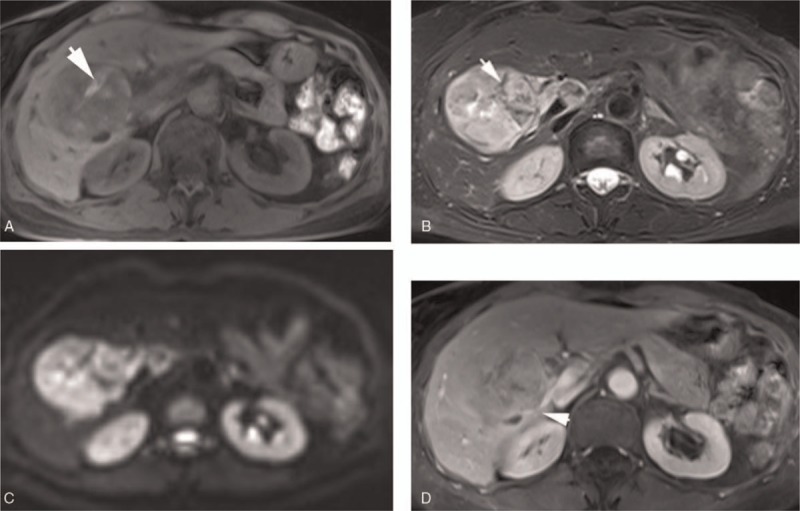
Magnetic resonance imaging revealed a heterogeneous mass in the hepatic hilar region. The mass was lobular and demonstrated expansive growth. (A) The mass displayed uneven low-signal intensity on T1-weighted imaging (T1WI) and (B) slightly high-signal intensity on T2-weighted imaging (T2WI). The strip in the tumor exhibited high-signal intensity on T1WI and low-signal intensity on T2WI (arrow), suggesting the existence of melanin. (C) Diffusion-weighted imaging revealed a restricted diffusion change, and (D) the tumor was unevenly enhanced after enhancement. Irregular liquefactive necrosis was discerned in the tumor. The tumor compressed the intrahepatic bile duct and caused dilatation and also invaded the right branch of the portal vein (arrow).

The image characteristics were as follows: CT revealed a circular low-density shadow of approximately 7.5 × 8.0 cm in the hepatic hilar region (Fig. 1). The interior of the mass was uneven, and high-density strips were observed in the mass. The tumor was slightly and unevenly enhanced in the arterial phase and further enhanced in the portal venous phase, and the interior of the mass was uneven. The tumor compressed the intrahepatic bile duct and caused dilatation, and tumor thrombus developed in the right branch of the portal vein. MRI revealed a heterogeneous mass in the hepatic hilar region, and the lobes were lobulated and expansive (Fig. 2). The mass exhibited heterogeneous low-signal intensity on T1-weighted imaging (T1WI) and slightly higher signal intensity on T1-weighted imaging (T2WI). Multiple irregular strips were identified in the tumor, which had high-signal intensity on T1WI and low-signal intensity on T2WI. DWI with b value 1000 appeared as a restricted diffusion change that enhanced both the slightly uneven enhancement of the mass in the arterial phase and continuous enhancement in the later stage. An area of irregular liquefactive necrosis was identified in the mass. The tumor compressed the intrahepatic bile duct and caused dilatation that invaded the right branch of the portal vein. The tumor in hepatic hilar region had an abundant blood supply. Bleeding and necrosis were observed inside the tumor. The tumor simultaneously invaded the portal vein and bile duct, and the common bile duct expanded. Observation indicated that the tumor was considered to be a malignant hepatic tumor. The MRI revealed heterogeneous high-signal intensity on T1WI and low-signal intensity on T2WI for multiple areas in the tumor, indicating the possible presence of melanin. Therefore, the tumor was suspected to be melanoma.

**Figure 3:**
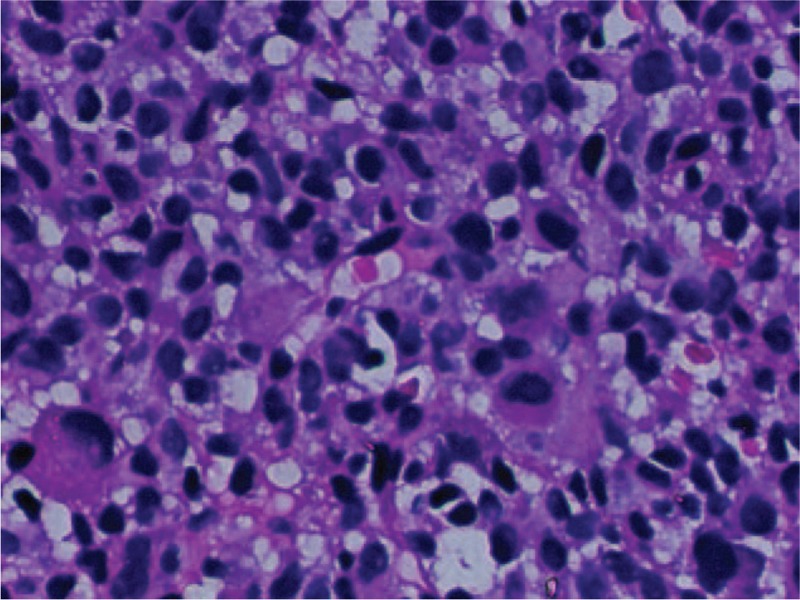
Hematoxylin and Eosin staining (×40), oval-shaped heterotypic cells demonstrated diffuse hyperplasia, and nucleoli were apparent. Pathological nuclear division was obvious, and scattered melanin particles were visible.

In September 2018, a biopsy was performed on the patient under local anesthesia, and the pathology of the liver biopsy sample suggested that the tumor was malignant. Furthermore, immunohistochemistry proved that the tumor was malignant melanoma (Fig. 3). The results of the immunohistochemical detection of the monoclonal antibody and oncogene revealed Vim(+), CD117(+), AFP(−), CD10(−), CEA(−), Glypican-3(−), Ki-67(+,50%), P53(+), CgA(−), SyN(−), CD56(+), PCK(−), HMB45(+), S-100(+), MelanA(+), CD34(+), CK7(−), CK19(−), CK18(−), CK8(−), CK20(−), CDX-2(−). Exhaustive examination of the skin, eyes, paranasal sinus, vulva, anus, genital tract, and gastrointestinal tract, and other parts was performed and no other possible primary site of malignant melanoma was detected. Then, the clinical diagnosis of PHM was made.

The tumor was located in the hepatic hilar region adjacent to the large blood vessels and bile duct, and the tumor invaded the portal vein. Because a resection was highly risky, conservative treatment was conducted. During hospitalization, the patient underwent symptomatic treatment, including anti-infection treatment, anemia correction, hypoproteinemia treatment, liver protection, stomach protection, and nutritional support.

**Figure 4:**
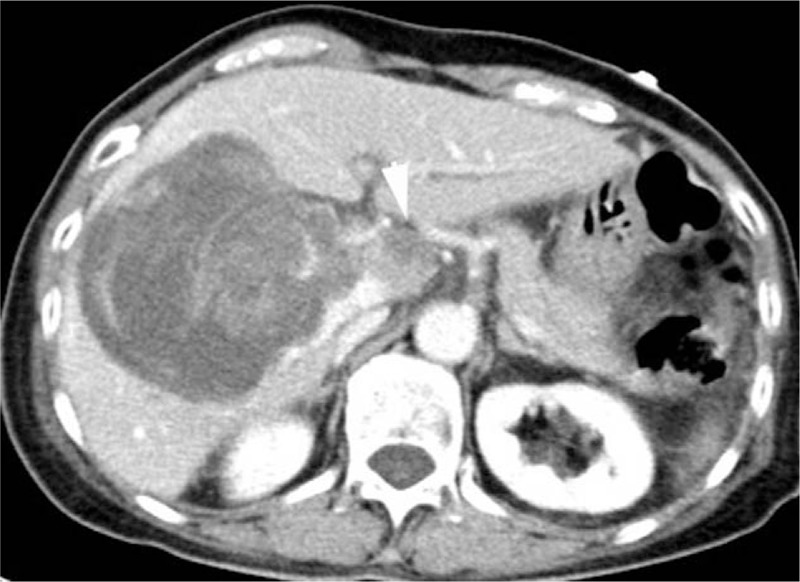
The computed tomography scan conducted after 2 months indicated a substantial increase in tumor size. The density of the tumor was uneven, and new lesions were discovered to be developing in the liver caudate lobe (arrow). The tumor invaded the blood vessels and bile ducts in the hepatic hilar region.

The patient has been followed up for 6 months. In November 2018, 2 months after conservative treatment, the patient underwent another CT examination. The results of the CT scan revealed that the tumor size had substantially increased (Fig. 4). A newly developed tumor appeared in the liver caudate lobe and invaded large blood vessels and bile ducts in the hepatic hilar region. In April 2019, the patient was reexamined by CT in another hospital. The results revealed peritoneal, omental metastases, and multi bone metastases.

## 3 Discussion

PHM is rare and has been insufficiently reported. The diagnosis of PHM is currently required to include the following three indicators: histopathologically confirmed diagnosis of PHM, exclusion of other types of primary malignant melanoma, and no unexplained skin tumors or surgeries to destroy or excise tumors without a histologic examination.^[9]^ The diagnosis of PHM primarily depends on clinical observation, imaging examination, and laboratory examination. The principal clinical symptoms are abdominal pain, abdominal mass, and weight loss. However, these clinical manifestations are not specific. This study summarizes the clinical features and imaging findings of this patient to serve as a reference for the preoperative diagnosis and differential diagnosis of PHM.

Imaging examination is essential for diagnosing melanoma. CT is the most commonly used method for diagnosing liver tumor lesions and is crucial for the localization and characterization of hepatic tumors. The soft-tissue resolution of MRI and its multiazimuth imaging are useful for evaluating intratumoral cystic changes and hemorrhagic necrosis. Multiphase dynamic imaging can be used to comprehensively investigate tumor enhancement patterns and characteristics.

Hepatic melanoma is primarily metastatic; PHM is extremely rare, and relevant studies are scarce. Only 4 studies related to PHM in English were identified in PubMed^[9–12]^ (Table 1). To our knowledge, the patient in this study was the first to have complete CT and MRI examination records and complete CT follow-up data.

**Table 1:**
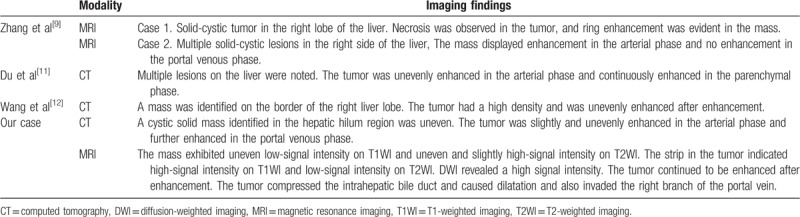
CT and MRI findings of PHM in relevant research and our case.

Zhang et al^[9]^ reported 2 PHM cases. MRI imaging revealed a 14 × 14 cm solid-cyst mass in right lobe of the first patient's liver. The mass exhibited high-signal intensity on T1WI and heterogeneous signal intensity on T2WI. Necrosis was observed in the center of the tumor, and ring enhancement was evident in the mass in the arterial phase and parenchymal phase. A solid-cyst of 14 × 12 cm in the right lobe of the liver was identified in the second patient by MRI. The lesion exhibited high-signal intensity on T2WI, the center of the lesion produced low-signal intensity on T2WI, and the corresponding area demonstrated high-signal intensity on T1WI. These results are all characteristic MRI signals of melanin in melanoma. Melanoma produces a paramagnetic substance, melanin; in MRI, melanin-containing melanoma indicates high-signal intensity on T1WI and low-signal intensity on T2WI, which is consistent with the results for the patient in the present study. Du et al^[11]^ presented a case study of PHM in which a CT scan revealed diffusion distribution of nodules of various sizes in the liver, unevenly enhanced lesions in the postarterial phase, and continuous enhancement of lesions in the portal phase and delayed phase scans accompanied by subtle ascites. Wang et al^[12]^ reported the details of a 12-year-old boy whose CT images indicated a low-density space-occupying mass of 5.9 × 4.2 cm in the right lobe of the liver. High-density shadows were observed in the center of the tumor, and the mass was uneven and enhanced after enhancement. The boundary was clear, and no lymph nodes or organ metastasis were observed.

In our case, CT imaging revealed a solid mass of round cysts in the hepatic hilar region that was unevenly enhanced after enhancement. The MRI results indicated a heterogeneous mass in the hepatic hilar region that exhibited homogeneous low-signal intensity on T1WI and slightly high-signal intensity on T2WI. Multiple irregular strips with high-signal intensity on T1WI and low-signal intensity on T2WI were discerned in the tumor. This study thus considered the tumor to contain melanin. A melanin-free region would produce equivalent or low-signal intensity on T1WI and equivalent or high-signal intensity on T2WI. However, the different melanin content in melanoma, differences in the tissue and organs affected by the lesions, and various factors (eg, bleeding and necrosis) all affect the diagnosis of abdominal melanoma and increase the difficulty of diagnosing abdominal melanoma using MRI imaging. Regarding the patient in the present study, DWI revealed a restricted diffusion change, and the tumor continued to exhibit uneven enhancement with internal necrosis. The tumor compressed the intrahepatic bile duct, caused dilatation, and invaded the right branch of the portal vein, which suggested that the tumor was invasive and that the patient had a poor prognosis. After 2 months, a CT scan indicated that the tumor had progressed, multiple new masses had appeared, the size of the mass had increased, and that the tumor had invaded the hepatic portal blood vessels and bile ducts. This study proposes the following features of PHM to assist diagnosis: single or multiple cystic-solid space-occupying lesions in the liver, expansive growth, possible capsule, easy bleeding, necrosis, and cystic lesion; after enhancement, the tumor may display flower-ring-shaped enhancement and no enhancement of the tumor in the central cystic necrosis area; the tumor manifests in various forms through MRI, that is, if the tumor exhibits high-signal intensity on T1WI and low-signal intensity on T2WI, then it may contain melanin. Moreover, such imaging results are the most characteristic of PHM.

In summary, this study presents a rare case study of PHM. The CT and MRI findings of PHM have rarely been reported in other studies. This study compiled a case study and literature review to define the imaging performance of PHM, which can serve as a reference for the preoperative diagnosis and differential diagnosis of PHM.

## Author contributions

**Conceptualization:** Guoqun Mao.

**Data curation:** Weiqun Ao, Jian Wang.

**Investigation:** Xiaoyu Han.

**Resources:** Guangzhao Yang, Xiaoyu Han.

**Supervision:** Guoqun Mao.

**Validation:** Yougen Cheng.

**Visualization:** Yuzhu Jia.

**Writing – original draft:** Weiqun Ao.

**Writing – review & editing:** Yougen Cheng.
